# Population pharmacokinetic modeling of intramuscular and oral dexamethasone and betamethasone in Indian women

**DOI:** 10.1007/s10928-020-09730-z

**Published:** 2021-01-03

**Authors:** Wojciech Krzyzanski, Mark A. Milad, Alan H. Jobe, Thomas Peppard, Robert R. Bies, William J. Jusko

**Affiliations:** 1 School of Pharmacy and Pharmaceutical Sciences, State University of New York, University of Buffalo, Buffalo, NY USA; 2 Milad Pharmaceutical Consulting LLC, Plymouth, MI USA; 3 Certara, Inc, Princeton, NJ USA; 4 Division of Pulmonary Biology, Cincinnati Children’s Hospital Medical Center, University of Cincinnati, Cincinnati, OH USA

**Keywords:** Betamethasone, Dexamethasone, Pharmacokinetics, Population modeling

## Abstract

Population analysis of pharmacokinetic data for five differing dosage forms and routes for dexamethasone and betamethasone in 48 healthy nonpregnant Indian women was performed that accounted for a partial and complex cross-over design. Single doses of 6 mg dexamethasone phosphate (DEX-P), betamethasone phosphate (BET-P), or 1:1 mixture of betamethasone phosphate and acetate (BET-PA) were administered orally (PO) or intramuscularly (IM). Plasma concentrations collected for two periods over 96 h were described with a two-compartment model with differing PO and IM first-order absorption inputs. Clearances and volumes were divided by the IM bioavailability $${F}_{IM}$$. The homogeneous ages, body weights, and ethnicity of the women obviated covariate analysis. Parameter estimates were obtained by the Laplace estimation method implemented in NONMEM 7.4. Typical values for dexamethasone were clearance ($${CL/F}_{IM})$$ of 9.29 L/h, steady-state volume ($${{V}_{ss}/F}_{IM})$$ of 56.4 L, IM absorption constant $$\left({k}_{aIM}\right)$$ of 0.460 1/h and oral absorption constant ($${k}_{aPO})$$ of 0.936 1/h. Betamethasone parameters were *CL/F*_*IM*_ of 5.95 L/h, $${Vss/F}_{IM}$$ of 72.4 L, $${k}_{aIM}$$ of 0.971 1/h, and $${k}_{aPO}$$ of 1.21 1/h. The PO to IM *F* values were close to 1.0 for both drugs. The terminal half-lives averaged about 7.5 h for DEX, 17 h for BET, and 78 h for BET from BET-PA with the latter reflecting very slow release of BET from the acetate ester. Overall, BET exhibited slower clearance, larger volume of distribution, faster absorption, and longer persistence than DEX. These data may be useful in considering exposures when substituting one form of corticosteroid for another.

**Supplementary information:**

The online version of this article (doi:10.1007/s10928-020-09730-z) contains supplementary material, which is available to authorized users.

## Introduction

Respiratory distress syndrome is the most significant factor contributing to morbidity and mortality of prematurely born neonates. High dose antenatal corticosteroids treatment of women at risk of preterm delivery is the standard treatment [1, 2]. Prolonged exposure to antenatal corticosteroids has been known to cause severe adverse effects for the fetus [3]. The therapeutic window for fetal corticosteroid plasma concentrations is unknown. Current recommended treatment by the World Health Organization comprises dexamethasone phosphate administered intramuscularly (IM) as four doses of 6 mg given at 12 h intervals, or betamethasone phosphate given IM as two doses of 12 mg given at a 24 h interval, or the one-to-one mixture of betamethasone phosphate and acetate as two IM doses of 12 mg given at a 24 h interval [4].

The pharmacokinetics (PK) of dexamethasone (DEX) in healthy volunteers is linear at clinically relevant dose ranges [5]. It is commonly administered as the sodium phosphate ester/salt and hydrolysis of the phosphate ester occurs within 1 h [5]. DEX is partly metabolized by CYP3A4 enzymes in the liver [6]. DEX clearance (*CL*) after IV dosing is about 0.18 L/h/kg and steady-state volume (*V*_*ss*_) is about 1.1 L/kg in healthy men [7, 8]. Betamethasone, the enantiomer of DEX, is also formulated as a sodium phosphate ester/salt that undergoes rapid hydrolysis after IV dosing yielding a BET *CL* of about 0.16 L/h/kg and *V*_*ss*_ of about 1.3 L/kg [9]. As with DEX, only a few percent of the BET dose is excreted unchanged in urine. Both DEX and BET bind moderately (about 65%) to plasma proteins [10]. While the PK of the IM BET phosphate/acetate mixture was well-characterized in sheep over a 5-day sampling showing rapid and slow/depot release components [11], previous PK studies in human subjects sampled blood only for limited periods and missed the complex PK and long terminal phase [12, 13]. These drugs exhibit increased clearances during pregnancy as described previously [10].

Our recent study of single doses of IM and PO DEX and BET plus the IM BET phosphate/acetate mixture employed blood stabilization of esters, 96 h blood sampling, and LC–MS/MS methodology to carefully assess the PK (and PD) of these steroid formulations in healthy Indian women [14]. However, the preliminary report was limited to a noncompartmental analysis (NCA) of the PK data. This report provides a population analysis of PK data for the five formulations in a partial and complex cross-over design in 48 healthy nonpregnant Indian women to seek further insights into the PK properties of these important therapeutic agents.

## Methods

### Study design

The study design was described previously [14]. It was an open-label, randomized, two-period study in healthy female subjects under fasting conditions. The subjects (N = 48) were randomized into eight sequences of 6 subjects who received two treatments during two periods separated by a washout time. The study design is presented in Table 1. Each period started with overnight fasting, followed by 24 h blood sampling for baseline biomarker measurements, the drug administration at 7 AM, and subsequent blood draws up to 96 h. The subsequent washout lasted 10 days. The study protocol was approved by the ACE Independent Ethics Committee, Bangalore, India and by the Institutional Review Board at Cincinnati Children’s Hospital Medical Center. Further details are available at ClinicalTrials.gov NCT03668860.

**Table 1:** The cross-over study design

Sequence	N	Period 1	Period 2
AB	6	DEX-P IM	BET-P IM
BA	6	BET-P IM	DEX-P IM
CD	6	BET-PA IM	DEX-P PO
DC	6	DEX-P PO	BET-PA IM
ED	6	BET-P PO	DEX-P PO
DE	6	DEX-P PO	BET-P PO
CE	6	BET-PA IM	BET-P PO
EC	6	BET-P PO	BET-PA IM

### Subjects

The female subjects in the study were of ages 22–39 years with normal body mass index 20.6–25.0 kg/m^2^. The ranges for height were 144–167 cm and weight 47.0–68.7 kg. All subjects were of Indian ethnicity. The study inclusion criteria ensured that the women were healthy, non-smokers or moderate smokers, non-drinkers or occasional drinkers, not pregnant, and using contraception. All subjects consented to participate in the study.

### Drug administration

All subjects were given single doses of 6 mg of either DEX or BET in each period as one of dosing groups A, B, C, D, and E. Description of treatments is provided in Table 2. There were 6 subjects in each treatment group and each subject received two treatments during the study. The cross-over sequences were AB, BA, CD, DC, ED, DE, CE, and EC. The sequence selection sought to obtain reasonable numbers of women in cross-over comparisons of each drug and formulation in the limited study population. The doses listed for PK were the free alcohol equivalents in the formulations. Drug content of all formulations was confirmed by LC–MS/MS analysis.

**Table 2:** Description of dosing groups in the study

Treatment	Route	Number of subjects who received treatment	Formulation
A	IM	12	Dexamethasone phosphate solution (Fresenius Kabi USA LLC)
B	IM	12	Betamethasone phosphate solution (BETENESOL®_,_ Glaxo SmithKline Pharmaceuticals Ltd, India)
C	IM	24	Betamethasone phosphate (3 mg) and acetate (3 mg) suspension (Celestone®, Merck and Co., Inc., USA)
D	PO	24	0.5 mg dexamethasone phosphate tablets (Cadila Healthcare Ltd, India)
E	PO	24	0.5 mg betamethasone phosphate tablets (BETNESOL®, Glaxo SmithKline Pharmaceuticals Ltd, India)

There were 6 subjects in each treatment group and each subject received two treatments. Total doses were all 6 mg

### Blood sampling

The time of drug administration for both periods was considered as a reference for the study time (t = 0). The blood samples for PK measurements were drawn at 0 (pre-dose), 0.5, 1.0, 1.5, 2.0, 3.0, 4.0, 6.0, 12, 18, 24, 30, 36, 48, 60, 72, and 96 h after dosing. Anticoagulant K_2_EDTA was added to blood samples and Na_2_HASO_4_ was added to prevent dephosphorylation of the DEX-P and BET-P phosphates in plasma. Plasma was separated from blood samples by centrifugation at 4 °C within 30 min after withdrawal. These samples were stored at − 70 °C before further analysis.

### Bioanalytical methods

The DEX and BET concentrations in plasma were quantified using validated LC–MS/MS methodology at Syngene Bioanalytical Research Laboratory (Syngene International Ltd, Bangalore, India). Dexamethasone-D4 and betamethasone-D4 were used as internal standards. The procedure involves initial solid phase extraction using the Orochem Technologies Ezypress 48, Ezypress HT 96 C, PTM 144 PPP Prochem Tech Machine method. The drugs and internal standards were separated on a Chiralpak IF-3, 150 × 4.6 mm, 3 μm column at 45 °C with a mobile phase consisting of acetonitrile:methanol (50:50 v/v) containing 0.05% formic acid:5 mM ammonium formate solution (90:10 v/v) at a flow rate of 1.0 mL/min using HPLC system Shimadzu Prominence (Shimadzu Nexera, Exion) The mass spectrometric detector was used with multiple reaction monitoring [dexamethasone: 437.200 → 361.100 (m/z), betamethasone: 437.200 → 361.100 (m/z), dexamethasone-D4: 441.200 → 363.100 (m/z) and betamethasone-D4: 441.200 → 363.100 (m/z)]. Each analysis required no longer than 5.0 min. The mass spectrometer was a Triple Quad™ 6500, API 6500 + MDS SciEx, (Applied Biosystems). Quantitation was achieved by measurement of the peak area ratios. The lower limit of quantification (LLOQ) of BET and DEX was 0.1 ng/mL. The inter-run precisions for both drugs at the LLOQ was less than 11%.

### Fixed effects model

**Fig. 1:**
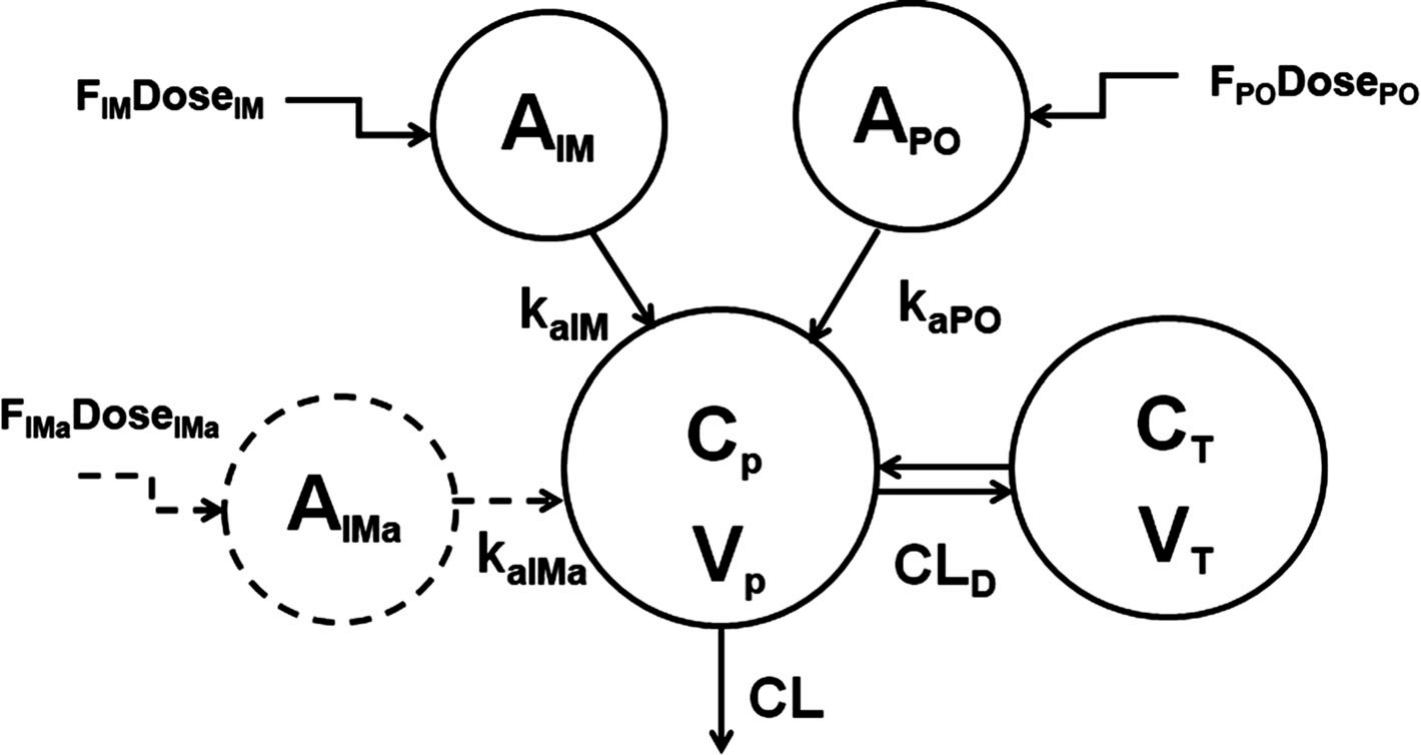
Pharmacokinetic model of DEX and BET following IM and PO administration. Kinetic processes are described by Eqs. (1)–(5) and model symbols are defined in the text and Tables 2 and 3

As both DEX and BET exhibited biexponential disposition [14], we adopted a two-compartment model with differing first-order absorption inputs for IM versus PO dosing. An additional absorption compartment was included to account for a potential difference in absorption rates between betamethasone phosphate and acetate for BET-PA. A schematic of the model reflecting both drugs is shown in Fig. 1. Model equations for the free alcohol forms of the drugs were:

1 $$ V_{p} \frac{{dC_{p} }}{dt} = k_{aIM} \cdot A_{IM} + k_{aPO} \cdot A_{PO} + k_{aIMa} \cdot A_{IMa} - \left( {CL + CL_{D} } \right) \cdot C_{p} + CL_{D} \cdot C_{T} $$

2 $$ V_{T} \frac{{dC_{T} }}{dt} = CL_{D} \cdot C_{p} - CL_{D} \cdot C_{T} $$

3 $$ \frac{{dA_{IM} }}{dt} = - k_{aIM} \cdot A_{IM} $$

4 $$ \frac{{dA_{PO} }}{dt} = - k_{aPO} \cdot A_{PO} $$

5 $$ \frac{{dA_{IMa} }}{dt} = - k_{aIMa} \cdot A_{IMa} $$

with the initial conditions:

6 $$ C_{p} (0) = 0,\,C_{T} (0) = 0,\,A_{IM} (0) = F_{IM} Dose_{IM} ,\,A_{PO} (0) = F_{PO} Dose_{PO} ,\,A_{IMa} (0) = F_{IMa} Dose_{IMa} $$

where corticosteroid central (*C*_*p*_) and peripheral tissue (*C*_*T*_) concentrations have corresponding volumes ${V}_{p}$ and ${V}_{T}$. The bioavailabilities *F*_*IM*_, *F*_*PO*_, and *F*_*IMa*_ indicate fractions of the dose absorbed via the first-order processes *k*_*aIM*_, *k*_*aPO*_*,* and *k*_*aIMa*_ from the PO and IM dosing compartments. Drug is cleared from the plasma with clearance *CL* and distributed to peripheral tissues with distributional clearance *CL*_*D*_. The disposition parameters *CL*, *CL*_*D*_, ${V}_{p}$ and ${V}_{T}$ are independent of route of administration and formulation. The dose values were 6 mg for DEX-P and BET-P. For BET-PA, ${Dose}_{IM}=3$ mg of BET phosphate, and ${Dose}_{IMa}=3$ mg of BET acetate. In the absence of IV dosing the bioavailability parameters are not identifiable. Therefore, the relative bioavailability parameter used ${F}_{IM}$ as a reference:

7 $$ F_{r} = \frac{{F_{PO} }}{{F_{IM} }}\,{\text{and}}\,F_{ra} = \frac{{F_{IMa} }}{{F_{IM} }} $$

The bioavailability ${F}_{IM}$ was combined with clearances $CL/{F}_{IM}$, ${CL}_{D}/{F}_{IM}$, and volumes ${V}_{p}/{F}_{IM}$, ${V}_{T}/{F}_{IM}$ to ensure identifiability of all model parameters.

Additional pharmacokinetic descriptors were peak plasma concentrations (*C*_*max*_) and peak time (*t*_*max*_), terminal half-life (*t*_*1/2*_), and Mean Residence Time (*MRT*) based on NCA analysis applied to the model-predicted individual *C*_*p*_ time courses. Individual *AUC* values were calculated as a solution to:

8 $$ \frac{dAUC}{{dt}} = C_{p} ,\quad AUC\left( 0 \right) = 0 $$

For this, individual ${C}_{p}$ and *AUC* values were simulated every hour up to 96 h. The *C*_*max*_ and *t*_*max*_ were then obtained from the maximum of the simulated data and *t*_*1/2*_ was calculated from the 95 and 96 h time points. The *MRT* was calculated as follows:

9 $$ MRT = \frac{{CL/F_{IM} }}{{V_{ss} /F_{IM} }} - \frac{1}{{k_{a} }} $$

where ${k}_{a}={k}_{aIM}\, \text{or} \,{k}_{aPO}$ for DEX and BET IM and PO administrations and ${k}_{a}={(Fr+{Fr}_{a})/(Fr/k}_{aIM}+{{Fr}_{a}/k}_{aIMa})$ for BET-PA IM administration.

### Random effects model

The interindividual variability (IIV) with the log-normal distribution was allowed for parameters:

10 $$ P = \theta_{P} \exp \left( {\eta_{P} } \right)\,\text{and}\quad\eta_{P} \sim {\mathcal{N}}\left( {0,\omega_{P}^{2} } \right) $$

where ${\theta }_{P}$ is a typical value of parameter $P$, ${\eta }_{P}$ denotes its individual realization, and ${\omega }_{P}^{2}$ is the variance in the log-domain. Selection of IIV parameters for the final model started with variances for $CL/{F}_{IM}$, ${V}_{p}/{F}_{IM}$ and increasing their number until either the estimation failed, precision of estimates were unacceptable, or goodness of fits plots were aberrant.

The observed drug plasma concentrations ${C}_{ij}$ were log-transformed and the constant residual error model was applied:

11 $$ \log C_{ij} = \log C_{p} \left( {t_{ij} } \right) + \varepsilon_{i} \,\,{\text{and}} \quad \varepsilon_{i} \sim {\mathcal{N}}\left( {0,\sigma^{2} } \right) $$

where ${t}_{ij}$ is the $j{\text{th}}$ observation time for the ${i}{\text{th}}$ subject and ${\sigma }^{2}$ is the variance of the residual error ${\varepsilon }_{i}$ that was same for all subjects.

### Parameter estimation and simulations

The DEX and BET data were fitted independently but data for all dosage forms for each steroid were fitted jointly. The observation times for sequences AB, BA, CD, DC, DE, and ED were considered as elapsed time since the last dose. For sequences CE and EC each subject received two doses of BET (BET-P PO and BET-PA IM). To avoid assigning two sets of times since the last dose to one subject, the observation times since the first dose were used. Additionally, for the sequence CE the BET-PA exhibited a prolonged terminal phase extending beyond the 10 day washout period that was manifested as residual BET plasma concentrations at pre-dose times for the next period. Therefore, for sequence CE the time since the first dose was a natural choice.

Model parameters were estimated by maximizing the likelihood of observations using the Laplace with Interaction method implemented in NONMEM 7.4 (ICON Clinical Research LLC, North Wales, PA), The data below the lower limit of quantification (LLOQ) were handled using the Beal M3 method for which the likelihood objective function is corrected by the probability of observations falling below the limit [15]. Evaluation of model performance were done by assessing change in the objective function value, standard errors of parameter estimates, goodness-of-fit plots, and visual predictive checks (VPC). The plots were obtained by R 4.0.0 packages (ggplot2, lattice, vpc) [16] using RStudio 1.1.383 [17]. Part of the NONMEM code is provided in the Supplemental Materials.

## Results

The total were 578 observations of which 103 were below the LLOQ for DEX, and 949 observations with 19 below the LLOQ for BET. There were 6 plasma concentrations that were above the LLOQ at the beginning of the second period following dosing with BET-PA IM. They averaged 0.35 ± 0.1 ng/mL.

**Fig. 2:**
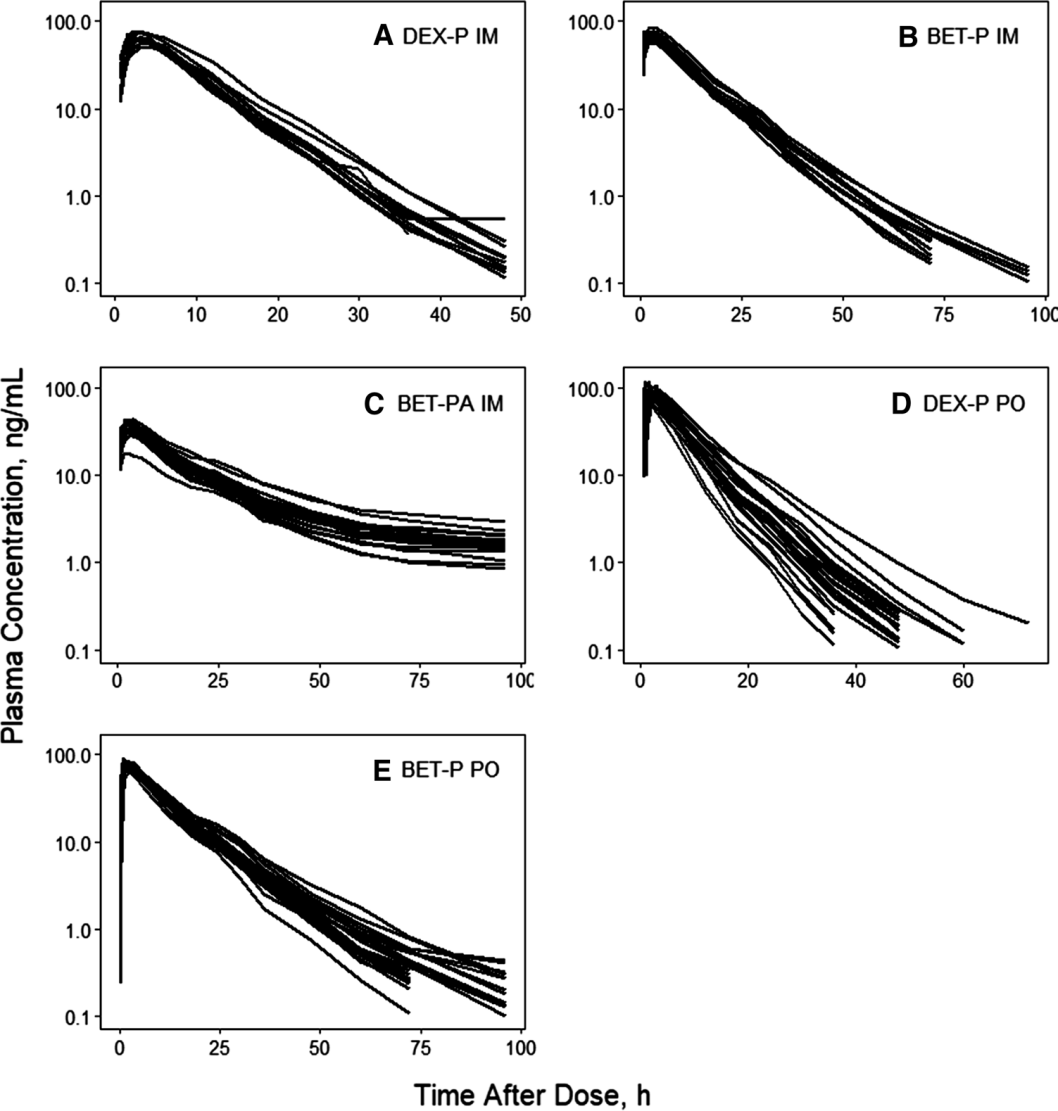
Spaghetti plots of individual subject DEX and BET plasma concentration time courses for indicated drugs and dosing routes

Spaghetti plots of individual time courses of DEX and BET plasma concentrations following each dosage form are shown in Fig. 2. The observed plasma concentrations in each dosing group are very tight. All curves showed at least two exponential decline phases. The DEX PO and BET PO profiles showed some spread at later times. The IM BET from BET-PA profiles exhibited a prolonged terminal phase owing to the slow hydrolysis/absorption from the acetate form. The early absorption phases showed fairly rapid, consistent, and smooth up-curves with rounded peaks. Interestingly, both DEX and BET exhibited earlier *t*_*max*_ values after PO rather than IM dosing. These properties supported the selection of the two-compartment model with the differing first-order absorption rates depending on the drug and route of administration.

We tested one- and two-compartment models with first-order absorption rates to describe DEX and BET PK data. Addition of a peripheral compartment significantly improved the fittings with a decrease in the objective function value 195.1 (DEX) and 379.5 (BET). The estimate of IIV for DEX apparent central volume was close to 0 and subsequently it was fixed at this value. We observed a correlation between individual estimates of BET $CL/{F}_{IM}$ and ${V}_{p}/{F}_{IM}$, so we additionally estimated their covariance. We were not able to estimate the covariance between $CL/{F}_{IM}$ and ${V}_{p}/{F}_{IM}$ for DEX with reasonable precision. We did not attempt to estimate IIV for the distribution parameters ${CL}_{D}/{F}_{IM}$ and ${V}_{T}/{F}_{IM}$ to avoid over-parameterization. We tested whether the presence of inter-occasion variability for *CL/F*_*IM*_ improved model performance in a subpopulation of subjects with sequences CE or EC. The drop in objective function value was 5.5, which implied, based on the likelihood ratio test, a significant improvement (p = 0.019) with no reduction of IIV or visible changes in other model diagnostics. Because this improvement was modest we decided not to include IOV in the final model for BET.

**Fig. 3:**
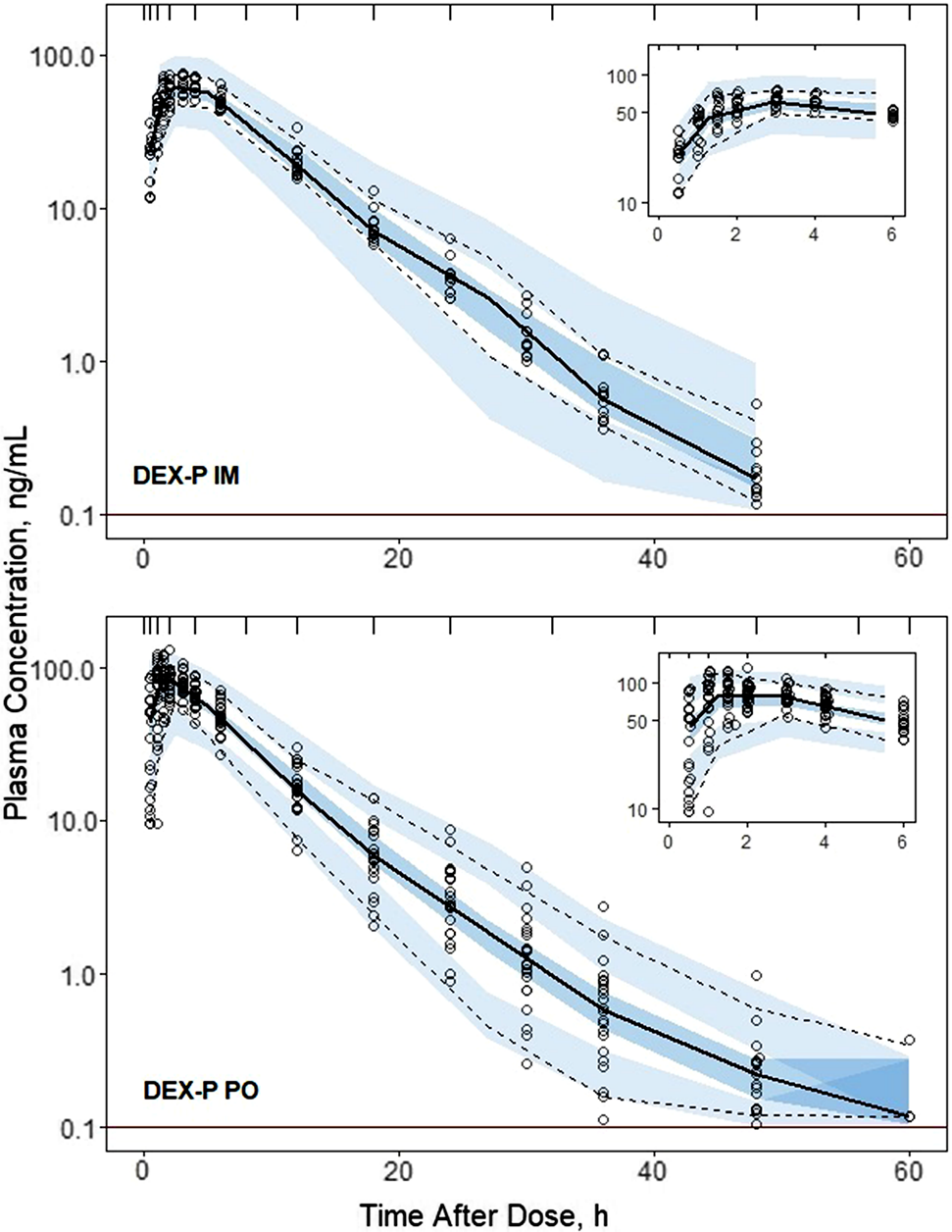
Visual predictive check plots for DEX following administration of 6 mg DEX-P IM (upper panel) and DEX-P PO (lower panel) doses. Symbols represent observed plasma concentrations, continuous line is the median, and dashed lines are 5th and 95th percentiles of observed values. The shaded regions are model-predicted confidence intervals for these percentiles. The insets show the plots over the first 6 h for better visualization. The horizonal line indicates the limit of quantitation (0.1 ng/mL)

**Fig. 4:**
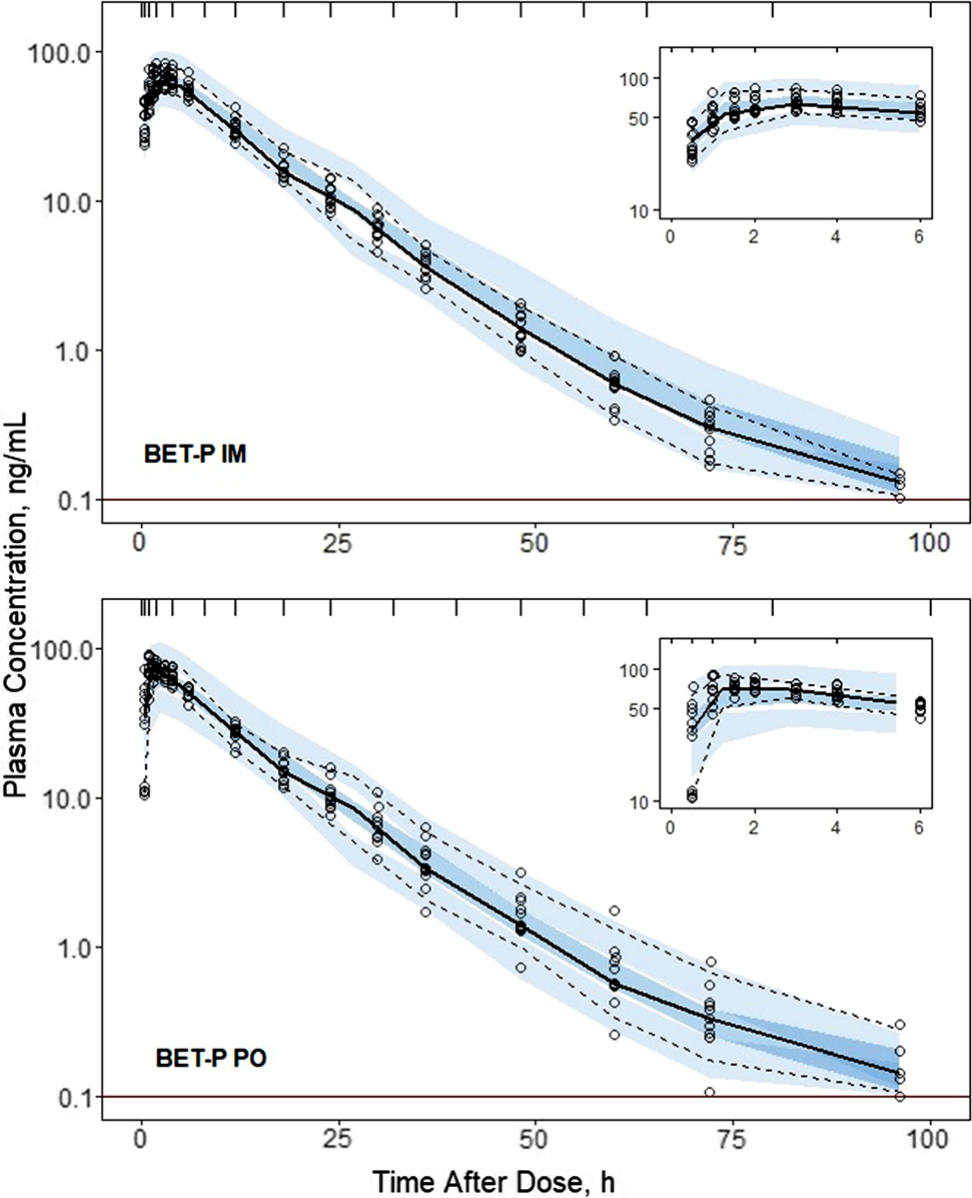
Visual predictive check plots for BET concentrations following administration of 6 mg BET-P IM (upper panel) and BET-P PO (lower panel) doses. Graph composition is the same as in Fig. 3

**Fig. 5:**
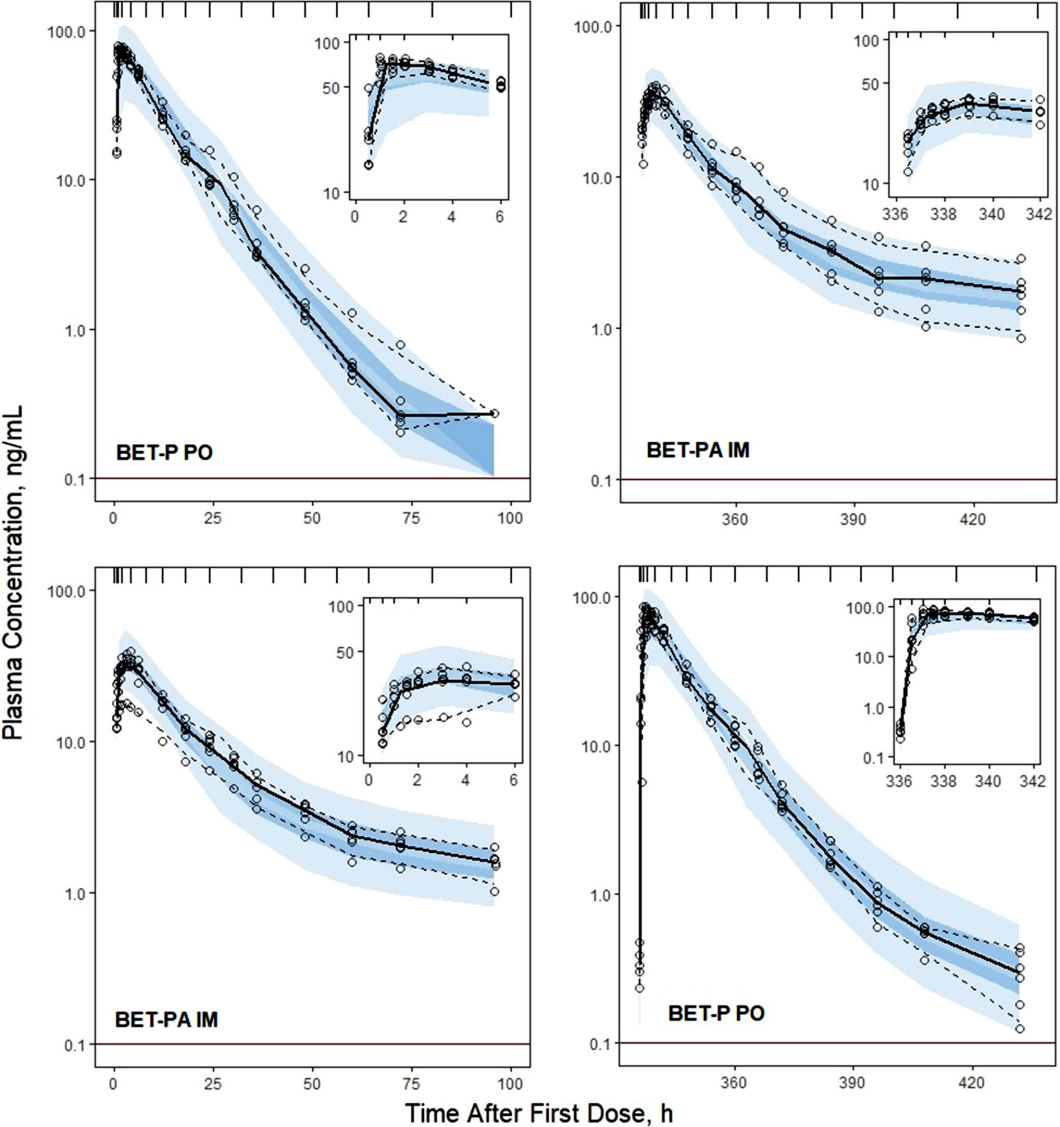
Visual predictive check plots for BET concentrations following administration of 6 mg BET-P PO and 6 mg BET-PA IM (upper panel), and 6 mg BET-PA IM and 6 mg BET-P PO (lower panel) doses. Graph composition is the same as in Fig. 3

**Fig. 6:**
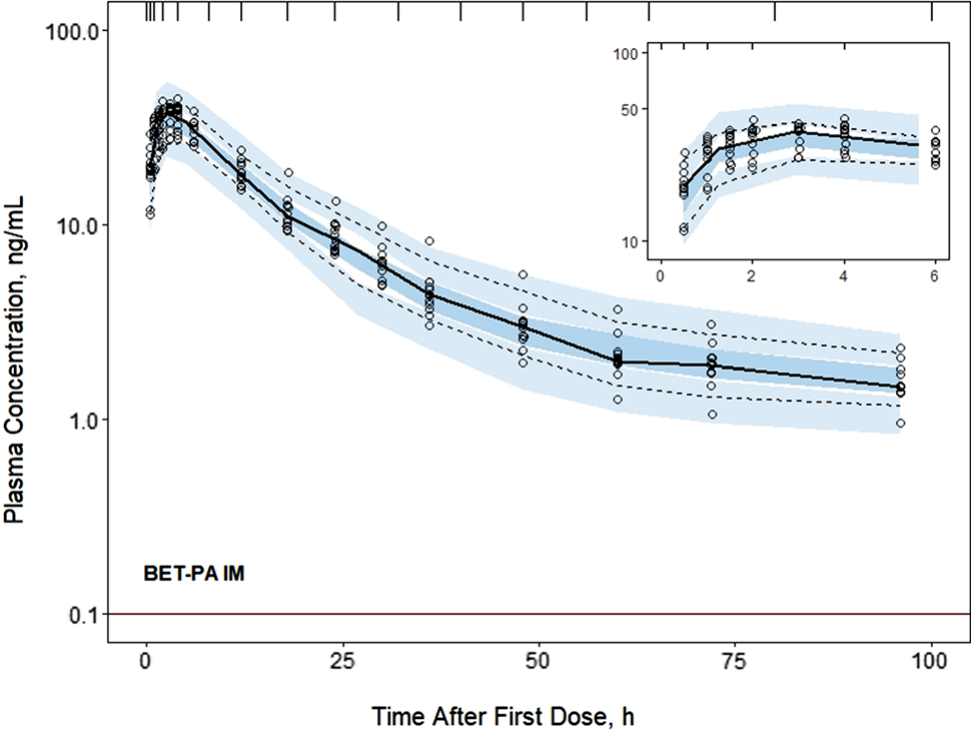
Visual predictive check plots for BET concentrations following administration of the 6 mg BET-PA IM dose. Graph composition is the same as in Fig. 3

Individual subject fittings of DEX and BET plasma concentrations versus time are shown in Supplementary Figures S1–S3. The individual profiles reinforce the patterns of the grouped profiles shown in Fig. 2 in terms of the rates of absorption, poly-exponential declines, and differences between DEX and BET. The very lengthy terminal phase from BET-PA extending to 14 days is evident in Figure S3. The model well described the observed data as seen in Figures S1–S3 and confirmed by the observed versus predicted diagnostic plots showing no systematic over- or under-predictions (Supplementary Figure S4). The VPC plots (Figs. 3, 4, 5, 6) demonstrate that the model captured the variability of data reasonably well with most parts of the medians and 5th and 95th percentile curves for observed values contained within the corresponding model-predicted confidence regions.

Estimates of population parameters for DEX and BET are presented in Tables 3 and 4. All typical values were estimated with good precision. The relative standard errors (%RSE) did not exceed 16%. The estimates of IIV parameters both for DEX and BET were moderately low with the highest variability for their PO absorption rates ${k}_{aPO}$. The low IIVs were confirmed by the VPCs shown in Figs. 3–6. These indicated slight over-prediction of plasma concentrations for subjects in the DEX-P PO group and under-predicted for the DEX-P IM group. The estimate of variance of ${F}_{ra}$ was small (Table 4) and warranted setting this parameter at 0 to reduce the number of model parameters. Since it still showed 8% IIV, it was kept as a model parameter. The %RSEs of estimates of IIV parameters for DEX were less than 16% (Table 3), while for BET the %RSE were less than 15% (Table 4) with the exception of the covariance between $CL/{F}_{IM}$ and ${V}_{p}/{F}_{IM}$.

**Table 3:** Dexamethasone population PK parameter estimates along with percent relative standard errors (%RSE)

Parameter	Definition	Estimate (%RSE) of typical value	Estimate (%RSE) of variance
*CL/F*_*IM*_, L/h	Apparent clearance	9.29 (4.4)	0.0265 (4.7) (16.4)*
*V*_*p*_*/F*_*IM*_, L	Apparent central volume	51.3 (4.4)	0**
*k*_*aIM,*_ 1/h	First-order absorption rate for IM administration	0.460 (8.8)	0.0633 (29.7) (25.6)*
*k*_*aPO*_, 1/h	First-order absorption rate for PO administration	0.936 (15.2)	0.395 (23.6) (69.6)*
*F*_*r*_	Relative bioavailability, *F*_*r*_ = *F*_*PO*_*/F*_*IM*_	1.04 (5.3)	NA
*CL*_*D*_*/F*_*IM*_, L/h	Apparent distributional clearance	0.538 (4.1)	NA
*V*_*T*_*/F*_*IM*_, L	Apparent volume of peripheral compartment	5.06 (4.7)	NA
*σ*^*2*^	Residual error	NA	0.0455 (18.9)

*Variance of log-normal distribution expressed as CV%

**Parameter value was fixed

**Table 4:** Betamethasone population PK parameter estimates along with percent relative standard errors (%RSE)

Parameter	Definition	Estimate (%RSE) of typical value	Estimate (%RSE) of variance
*CL/F*_*IM*_, L/h	Apparent clearance	5.95 (8.6)	0.0210 (13.9) (14.6)*
*V*_*p*_*/F*_*IM*_, L	Apparent central volume	67.5 (3.2)	0.0188 (10.7) (13.8)*
*k*_*aIM,*_ 1/h	First-order absorption rate for IM administration	0.971 (3.6)	0.0441 (7.9) (21.2)*
*k*_*aIMa*_, 1/h	First-order absorption rate for BET acetate after IM administration	0.00638 (14.7)	0.147 (2.1) (39.8)*
*k*_*aPO*_, 1/h	First-order absorption rate for PO administration	1.21 (1.5)	0.241 (0.8) (52.2)*
*F*_*r*_	Relative bioavailability, *F*_*r*_ = *F*_*PO*_*/F*_*IM*_	0.935 (9.7)	0.0182 (8.1) (13.6)*
*F*_*ra*_	Relative bioavailability, *F*_*ra*_ = *F*_*IMa*_*/F*_*IM*_	0.819 (9.1)	0.00773 (0.02) (8.8)*
*CL*_*D*_*/F*_*IM*_, L/h	Apparent distributional clearance	0.173	NA
*V*_*T*_*/F*_*IM*_, L	Apparent volume of peripheral compartment	4.94	NA
*Cov(CL/F*_*IM*_, *V*_*p*_*/F*_*IM*_)	Covariance of *CL/F*_*IM*_ and *V*_*p*_*/F*_*IM*_	NA	0.0155 (37.2) (0.78)*
*σ*^*2*^	Residual error	NA	0.0211 (0.03)

*Variance of log-normal distribution expressed as CV%

The derived secondary PK descriptors for the five drug dosing groups are listed in Supplementary Table S1. The *C*_*max*_*, t*_*max*_*,* and *t*_*1/2*_ were calculated for individual predicted plasma concentrations. The mean of model-predicted individual *C*_*max*_ values were similar as 62.5 for DEX-IM and 78.9 ng/mL for DEX-PO. The corresponding individual peak times *t*_*max*_ were 3.3 and 2.2 h, and terminal half-lives *t*_*1/2*_ were 7.5 and 7.6 h. The *C*_*max*_ values were very close as 66.9 for BET-IM and 65.9 ng/mL for BET-PO with corresponding *t*_*max*_ values of 2.8 and 2.6 h, and *t*_*1/2*_ values of 14.9 and 18.7 h. For IM BET-PA the *C*_*max*_ was 35.8 ng/mL, *t*_*max*_ was 2.9 h, and *t*_*1/2*_ was 77.6 h. These values are generally similar to those obtained previously either by NCA or by inspection of the data [14], but the latter approach is influenced by the sampling times that were selected.

The differences in PO and IM first-order absorption rates for DEX and BET are interesting. Oral absorption was faster with an absorption half-life of 0.74 h for DEX and 0.57 h for BET. The IM absorption half-lives averaged 1.51 h for DEX and 0.71 h for BET. It was assumed in the modeling that the absorption rate of BET-IM also applied to the phosphate form in BET-PA, which was supported by the good capture of the early part of the profiles for BET-PA (Figure S3). The hydrolysis/absorption half-life for the BET acetate component was 109 h, indicating that ‘flip-flop’ kinetics control the terminal phase of the BET-PA curves.

**Fig. 7:**
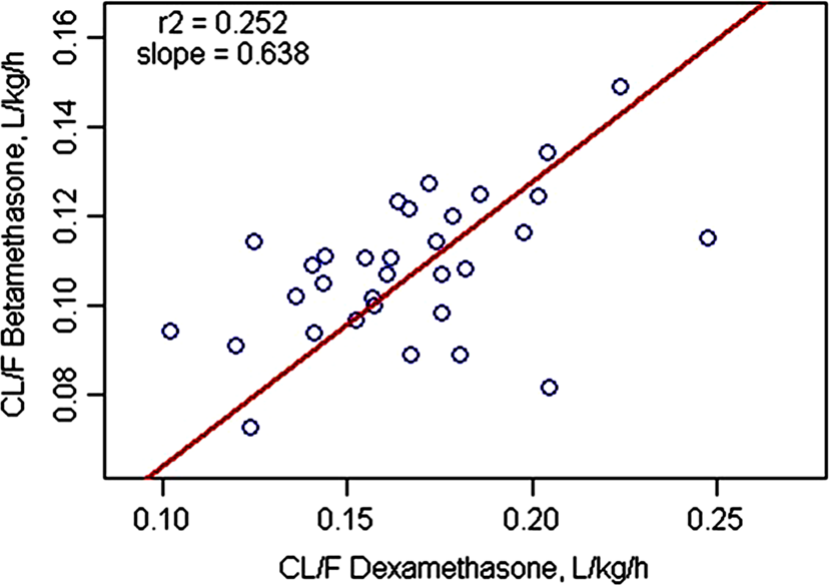
Correlation of model-derived clearance values for DEX versus BET in N = 32 individual subjects who received DEX-P and BET-P

Many of the subjects received both DEX and BET as part of the cross-over design and thus it was possible to compare the model-fitted $CL/{F}_{IM}$ values in 32 women as shown in Fig. 7. The values correlate weakly with r^2^ = 0.252. The slope, as obtained by orthogonal least-squares regression, was 0.638 indicating that BET $CL/{F}_{IM}$ was generally about 64% of DEX $CL/{F}_{IM}$ in the same women. Counter-intuitively, the correlation was probably weak because the subjects were very similar healthy women without marked differences in metabolic rates (about twofold range for each drug) and thus small differences become exaggerated.

When normalized for the mean body weight of 56.8 kg, the mean $CL/{F}_{IM}$ was 0.16 for DEX and 0.10 L/h/kg for BET. The *V*_*ss*_/*F*_*IM*_ values (*V*_*p*_/*F*_*IM*_ + *V*_*T*_/*F*_*IM*_) averaged 0.99 for DEX and 1.28 L/kg for BET. These values (functioning as *CL*/*V*_ss_) largely determine the much longer *t*_*1/2*_ and MRT for BET.

**Fig. 8:**
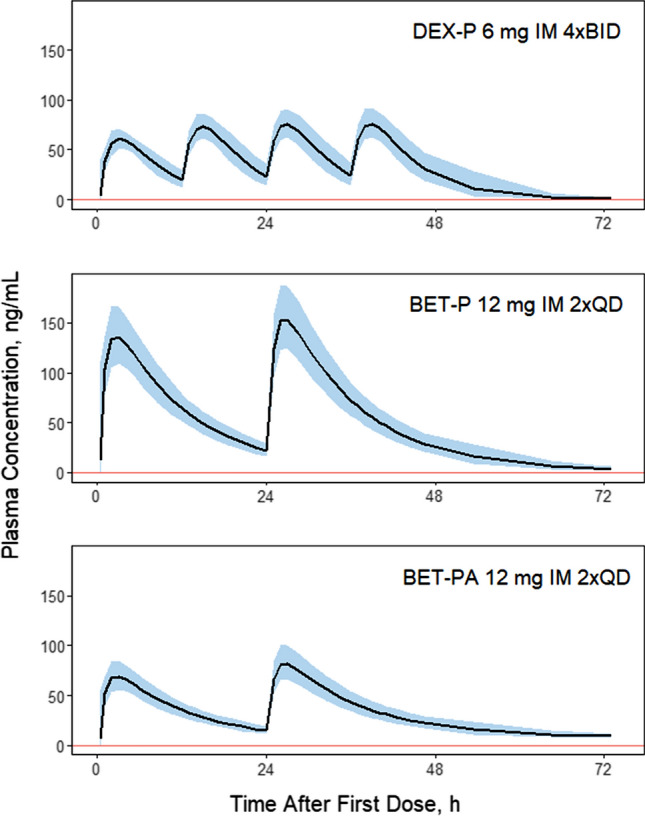
Simulated corticosteroid plasma concentrations for indicated dosing regimens. The solid line is the median of N = 200 subjects. The shaded regions represent 5th and 95th percentiles. The vertical line denotes the limit of quantification. Parameters used for simulations are listed in Tables 3 and 4

The estimates of the population parameters were used to simulate DEX and BET plasma concentrations for three clinically utilized dosing regimens for antenatal corticosteroid treatment: DEX-P as four doses of 6 mg given at 12 h intervals (6 mg IM BIDx4), BET-P as two doses of 12 mg given at a 24 h interval (12 mg IM QDx2), and BET-PA as two doses of 12 mg given at a 24-h interval (12 mg IM QDx2). The simulated PK profiles for these three regimens are shown in Fig. 8. The corresponding *C*_*max*_, *C*_*trough*_, and *AUC* values are listed in Supplementary Table S2. Both rapidly-absorbed forms of DEX and BET are eliminated after 72 h, but the BET-PA formulation exhibits significantly prolonged concentrations in plasma. The peak and *AUC* values are similar for DEX-P and BET-PA whereas the *C*_*max*_ is twice and *AUC* is 1.5-fold higher for BET-P.

## Discussion

This study and analysis were enacted for multiple reasons. Both DEX and BET are used and being further studied for treatment of women at risk of pre-term delivery, with IM dosing as the recommended route of administration [4]. Although a previous assessment used physiologically-based PK modeling to compare the two steroids [10], this study was the first that we could identify to provide a head-to-head comparison of their PK in a direct cross-over study in human subjects, particularly reproductive-age women [14]. Secondly, the potential exists for use of either oral DEX or BET in the absence of availability of the injectable dosage forms. Thus, the comparison of the oral route of administration is of interest. The PK parameters for these drugs will be used in further comparison of several pharmacodynamic (PD) biomarkers including cortisol suppression and cell trafficking in the same volunteer subjects [14]. Notably, DEX and BET are enantiomers with the same logP, protein binding, and presumably absorption and metabolic pathways [10] and it is intriguing whether there are PK and PD differences for these important therapeutic agents. Lastly, the PK of BET from BET-PA had not been fully characterized in humans previously.

Previous studies of the PK/PD of DEX and BET were designed to assess these drugs individually, although the PK/PD of DEX has been compared to prednisolone and methylprednisolone in cross-over studies [18]. The partial cross-over design of our study gave us an opportunity to compare the PK/PD of these corticosteroids in the same subjects allowing assessment due to substantial reduction in inter-individual variability. Owing to the expected prolonged disposition and actions of the IM BET-PA and the greater sensitivity of the LC–MS/MS methodology compared to older RIA and HPLC methods [5, 7–9], the blood sampling was extended to 96 h after dosing. This allowed us to more accurately and definitively quantify the terminal phases of the poly-exponential disposition of DEX and BET following absorption after IM and PO administration. In fact, the 10 day washout period and cross-over design allowed us to detect BET after 14 days (Fig. 3S) and include such values in fittings of the BET-PA data. With the cross-over design of the study for BET, we assessed the sequence or period effect on PK and found a small improvement in the IIV. The PK profiles of the BET dosage forms were jointly well-fitted without this consideration.

The PK of both DEX and BET for each dose and route was very similar in each group of subjects (Fig. 2) with consistent profiles in individual subjects (Figures S1-S3) leading to very small estimates of variability parameters for absorption rate constants, clearances, and volumes (Tables 3 and 4). The fitting diagnostics (Supplemental Materials) show excellent capture of the experimental data with the models. It was not feasible to improve these model fittings with alternative kinetic features and the close similarity of the study subjects in terms of ages, weights, and ethnicity precluded including covariates. For example, the correlations of individual *CL/F*_*IM*_ values with body weight were r^2^ = 0.005 for DEX and r^2^ = 0.003 for BET.

Since both BET and DEX were administered IM and PO, the estimates of volumes and clearances are apparent. However, these compounds are dosed as highly-soluble ester/salts, DEX and probably BET fall in the Biopharmaceutics Classification System Class I as highly soluble and highly permeable [19], the relative *F* of PO to IM phosphate forms were essentially 1.0 (Tables 3 and 4), clearances are low offering very little first-pass metabolism, and thus it is likely that absorption was high or complete. Our clearance estimates are very similar to the values calculated using NCA for the same subjects of 6.5 L/h for BET and 9.5 L/h for DEX [14]. However, our $CL/{F}_{IM}$, for BET was 50% of the value reported for healthy volunteers after IV injection of 10.7 L/h [9]. The analogous typical value for DEX was 57% of 16.3 L/h [8]. Another reported value of $CL/{F}_{IM}$, for DEX in healthy male subjects was 18.2 L/h [18]. The difference between our estimates of $CL/{F}_{IM}$ and others may be related to the previous shorter durations of earlier PK studies that ranged 8–24 h compared to 96 h in our study. The lowest observed plasma concentrations in those studies were above 1 ng/mL, while our terminal phases of DEX and BET fell below that value. While one previous report showed no sex difference in PK of DEX [8], our female subjects likely had smaller body weights than male subjects in other studies. An ethnic difference cannot be ruled out, but an assessment of midazolam *AUC* values in Caucasian versus South Asian men showed a 27% higher clearance of this CYP3A4 compound in Asians [20]. However, DEX is partly metabolized by CYP3A4 in liver [6] and partly by 11β-hydroxysteroid dehydrogenase in kidney [21].

Similar study considerations pertain to comparison of volumes of distribution. The typical values of apparent *V*_*ss*_/*F*_*IM*_ estimated in our modeling were 72.4 L for BET and 56.4 L for DEX. The NCA-calculated values were 94.6 and 71.0 L [14]. The BET *V*_*ss*_ reported was 84 L [9]. The DEX *V*_*ss*_ values have been reported as 55 L [8] and 41.6 L [18].

The mean terminal half-lives were very similar for IM and PO dosing both for DEX and BET, consistent with the NCA values [14]. However, our estimates were about twice the values obtained for healthy subjects receiving DEX-P and BET-P as IV injections. These previous *t*_*1/2*_ values were 2.37 h for DEX [8] and 5.58 h for BET [9]. As in the case for *CL*/*F*_*IM*_*,* we attribute these differences to the shorter duration of these studies that were unable to capture a true terminal phase of elimination. The *t*_*1/2*_ = 12.9 h reported for healthy subjects who received BET-PA IM was calculated from blood samples taken only up to 48 h [12]. Our estimate of the mean half-life of BET from BET-PA of 77.6 h is sixfold longer with the profiles showing a well-captured terminal phase (Figure S3). For drugs with absorption phases and poly-exponential disposition, the *MRT* provides a better descriptor for mean duration of drug exposure than *t*_*1/2*_. The *MRT* values in Supplementary Table S1 show much longer values for all forms of BET compared to DEX, with the former about twice that of the latter.

An interesting difference in the terminal slopes of plasma concentration profiles between PO and IM subjects receiving DEX-P (and to a lesser degree BET-P) can be observed in Fig. 2. This difference resulted in slight overprediction and underprediction of IIV in the VPC plots for these groups (Fig. 3). Since these subjects differed only by route of administration, variability of terminal slopes is not likely to be explained by variability in clearances and volumes. Possibly some subjects exhibited flip-flop kinetics where the absorption process controls the terminal phase of plasma concentration profiles. However, an absorption time extending beyond 24 h is unlikely. In studies in rats, we reported an unusually strong avidity of DEX for liver that might result in greater uptake and slower release from the liver tissue following oral compared to IM dosing [22].

Our values for the central volumes of distribution (*V*_*p*_/*F*_*IM*_) of BET and DEX are relatively large, especially compared to IV doses, while the peripheral volumes are fairly small. The large *V*_*p*_/*F*_*IM*_ can be attributed to ‘disappearance’ of the true central or even plasma volume owing to the absorption phase of these drugs. Known as ‘vanishing exponentials’, an absorption phase can hide an early IV phase for poly-exponential disposition [23]. On the other hand, our *V*_*T*_/*F*_*IM*_ value reflects a new finding of a small “deep compartment” that was detected using the extended sampling times and high sensitivity assay. Prolonged sampling has demonstrated a similar late terminal phase after IV DEX dosing in horses [24].

Our estimate of a typical value of *k*_*a*_ for IM DEX-P was 0.46 1/h, a half-life of 1.51 h. One study of the IM injection of BET-PA in pregnant women yielded *k*_*a*_ = 3.1 1/h based on very sparse sampling [13], whereas our analysis yielded fast and slow absorption components. Our model and data are also consistent with dual absorption findings for this dosage form in sheep where the early absorption *t*_*1/2*_ was 0.3 h and the later one 13.2 h [11]. Previous studies report a *t*_*max*_ for PO DEX-P at about 1.4 h [7] and IM DEX-P at about 1.8 h [25]. Absorption of both DEX-P and BET-P after PO ingestion was faster than after IM injection. It is likely that the highly soluble sodium phosphate ester/salts dissolve more quickly in the GI fluids and are exposed to a greater surface area for absorption compared to the IM dosage forms given in a concentrated solution of 4 mg/mL. Estimates of BET *k*_*a*_ after IM injections of BET-P, allowed us to estimate the separate absorption/hydrolysis rate of BET from BET acetate at the IM injection site. Our estimate of *k*_*a*_ = 0.00638 1/h is consistent with a very slow dissolution/hydrolysis/absorption process that is responsible for the prolonged terminal half-life of BET *t*_*1/2*_ = 77.6 h and reflecting flip-flop kinetics.

The PK data for CS in pregnant women reported in literature are limited. Moreover, most of older data are based on analytical assays that were not sensitive at the low concentrations. The majority of publications report only noncompartmental PK parameters. In a recent clinical study, 103 pregnant women received 11.4 mg of BET-PA IM QDx2 [26]. A two-compartment model with first-order absorption was used for population analysis. The typical values for a 70 kg subject were *CL/F* = 11.6 L/h, *V*_*p*_*/F* = 140 L*, CL*_*D*_*/F* = 4.54 L/h, *V*_*T*_*/F* = 159 L*,* and *k*_*a*_ = 1.17 h^−1^. While the first two parameters are approximately twice higher than our estimates for BET, the *CL*_*D*_*/F* and *V*_*T*_*/F* are about 30-fold higher than our values for non-pregnant women. The absorption rate constant is similar. Pregnancy and fetal distribution may account for part of these differences.

The customary antenatal corticosteroid dosing regimens for DEX and BET aim at maintaining therapeutic exposures while minimizing the putative toxic peaks using the maternal plasma concentrations as a reference. We performed simulations of the three antenatal corticosteroid regimens recommended by the WHO [4] to compare their PK profiles. The regimen with BET (12 mg IM QDx2) showed similar troughs to the DEX regimen (6 mg IM BIDx4), the peak concentrations and *AUC* values were almost twice higher (Supplementary Table S2). A profile for BET-PA (12 mg IM QDx2) had the *C*_*max*_ values similar to ones for DEX-P, but the *C*_*trough*_ and *AUC* values were lowest of all three regimens. These profiles need further interpretation in relation to changes in PK during pregnancy [10, 27, 28] and comparative fetal distribution, efficacies, and adverse effects of the two steroids in pregnant women.

It can be pointed out that DEX is currently used at doses of 6 mg/day for treatment of patients with COVID-19 [29]. Our assessment of the PK indicates that use of DEX as either oral or IM dosage forms should produce very similar exposures (*C*_*max*_ and *AUC*) in these patients.

In summary, we performed population analysis of PK data for DEX and BET for PO and IM dosage forms in nonpregnant women that extends the results of the NCA published recently (14). The PK parameters are considered more definitive than earlier studies owing to blood sample stabilization, the repeated assessments, intensive and extended duration of blood sampling, high sensitivity of the LC–MS/MS assay, and use of state-of-the-art pharmacometric data analysis methods. Simulations of three clinically relevant dosing regimens for DEX and BET based on these data showed that BET-PA provides the lowest overall maternal exposures. Finally, our estimates of individual PK parameters will be applied to a population PKPD model of DEX and BET effects on cortisol, glucose, and cell trafficking responses in the studied subject population [14].

## Supplementary information

Below is the link to the electronic supplementary material.

Electronic supplementary material 1 (PDF 75 kb)

Electronic supplementary material 2 (PDF 1210 kb)
