# Supplementary material for: Population pharmacokinetic modeling of intramuscular and oral dexamethasone and betamethasone in Indian women
Source: J Pharmacokinet Pharmacodyn. 2021 Jan 3;48(2):261–72. doi: 10.1007/s10928-020-09730-z (PMC7778726; doi:10.1007/s10928-020-09730-z)
Supplement: Supplementary file 1 — Electronic supplementary material 1 (PDF 75 kb) [file 10928_2020_9730_MOESM1_ESM.pdf]

## Supplemental Material Code

# Population Pharmacokinetic Model of Intramuscular and Oral Dexamethasone and Betamethasone in Indian Women

**Wojciech Krzyzanski, Mark A Milad, Alan H Jobe, Thomas Peppard, Robert M Bies, William J Jusko**

### NONMEM control stream for BET with an excerpt from a data set

```
$PROB BETAMATHASONE PK
$INPUT ID TIME AMT CMT CONC DV EVID MDV BLQ PER SEQ BW
$DATA  C:\BET.CSV IGNORE=C
$SUBROUTINES ADVAN13 ATOL=12 TOL=12
$MODEL NCOMPARTMENTS=5
COMP=(IM)
COMP=(PLASMA)
COMP=(PERI)
COMP=(PO)
COMP=(IMA)
$PK
   CL       = THETA(1)*EXP(ETA(1))
   V        = THETA(2)*EXP(ETA(2))
   KAIM     = THETA(3)*EXP(ETA(3))
   KAIMA    = THETA(4)*EXP(ETA(4))
   KAPO     = THETA(5)*EXP(ETA(5))
   FR       = THETA(6)*EXP(ETA(6))
   FRA      = THETA(7)*EXP(ETA(7))
   CLD      = THETA(8)
   VT       = THETA(9)
   F4 = FR
```

```
   F5 = FRA
;INITIAL CONDITIONS
   A_0(1)  = 0
   A_0(2)  = 0
   A_0(3)  = 0
   A_0(4)  = 0
   A_0(5)  = 0
$DES
   DADT(1)  = - KAIM*A(1)
   DADT(2)  =   KAIM*A(1)+KAPO*A(4)+KAIMA*A(5)-CL/V*A(2)-
                CLD/V*A(2)+CLD/VT*A(3)
   DADT(3)  =   CLD/V*A(2)-CLD/VT*A(3)
   DADT(4)  = - KAPO*A(4)
   DADT(5)  = - KAIMA*A(5)
$ERROR
  IPRED=-10
  IF (CMT.EQ.2) IPRED= LOG(A(2)/V)
  SD=SQRT(SIGMA(1))
  LLOQ=LOG(0.1)
  IF (BLQ.EQ.0) THEN
   F_FLAG=0
   Y=IPRED + EPS(1)
  ENDIF
  IF (BLQ.EQ.1) THEN
   F_FLAG=1
   Y=PHI((LLOQ-IPRED)/SD)
  ENDIF
$EST METHOD=COND INTERACTION LAPLACIAN NUMERICAL SLOW PRINT=1
MAX=9999 SIG=3 NOABORT
```

```
$THETA
  $THETA
  (0,5.95 , )              ;1. L/h CL
  (0,67.5, )               ;2. L   V
  (0,0.971, )              ;3. 1/h KAIM
  (0,0.00638, )            ;4. 1/h KAIMA
  (0,1.21, )               ;5. 1/h KAPO
  (0,0.935, )              ;6. FR
  (0,0.819, )              ;7. FRA
  (0,0.173,)               ;8. L/h CLD
  (0,4.94,)                ;9. L   VT
$OMEGA BLOCK(2)
  0.021         ;1. CL
  0.0155        ; COV(CL,V)
  0.0188        ;2. V
$OMEGA
  0.0441        ;3. KAIM
  0.147         ;4. KAIMA
  0.241         ;5. KAPO
  0.0182        ;6. FRA
  0.00773       ;7. FRA
$SIGMA
  0.0211
$COV PRINT=E SLOW

$TABLE ID TIME CMT MDV IPRED CIWRES SEQ PER
C ID TIME   AMT     CMT  DV      EVID  MDV   BLQ  PER  SEQ  BW
2    0      6000    1    .       1     1     0    1    21   54.7
```

| | | | | | | | | | | |
|---|---|---|---|---|---|---|---|---|---|---|
| 2 | 0.0001 | . | 2 | -2.3035 | 0 | 1 | 1 | 1 | 21 | 54.7 |
| 2 | 0.5 | . | 2 | 3.2742 | 0 | 0 | 0 | 1 | 21 | 54.7 |
| 2 | 1 | . | 2 | 3.8296 | 0 | 0 | 0 | 1 | 21 | 54.7 |
| 2 | 1.5 | . | 2 | 3.9077 | 0 | 0 | 0 | 1 | 21 | 54.7 |
