# Supplementary material for: Population pharmacokinetic modeling of intramuscular and oral dexamethasone and betamethasone in Indian women
Source: J Pharmacokinet Pharmacodyn. 2021 Jan 3;48(2):261–72. doi: 10.1007/s10928-020-09730-z (PMC7778726; doi:10.1007/s10928-020-09730-z)
Supplement: Supplementary file 2 — Electronic supplementary material 2 (PDF 1210 kb) [file 10928_2020_9730_MOESM2_ESM.pdf]

Supplemental Material Tables and Figures

Population Pharmacokinetic Model of Intramuscular and Oral Dexamethasone and Betamethasone in Indian Women

Wojciech Krzyzanski, Mark A Milad, Alan H Jobe, Thomas Peppard, Robert M Bies, William J Jusko

Table 1S. Secondary pharmacokinetic descriptors**.** The C_{max}, t_{max}, and $t_{1/2}$ were calculated for individual predicted plasma concentrations. Data are presented as mean ±SD of individual estimates for each dosing group

Treatment	C_{max}, ng/mL	t_{max}, h	$t_{1/2}$, h	MRT, h
DEX-P IM	62.5 ±6.1	3.3 ±0.5	7.5 ±0.1	7.81 ±0.57
DEX-P PO	78.9 ±13.5	2.2 ±0.8	7.6 ±0.3	6.91 ±1.20
BET-P IM	66.9 ±8.4	2.8 ±0.4	14.9 ±0.7	12.1 ±0.74
BET-P PO	65.9 ±7.9	2.6 ±0.8	18.7 ±6.8	12.5 ±1.13
BET-PA IM	35.8 ±4.7	2.9 ±0.4	77.6 ±14.3	96.5 ±33.2

Table 2S. Simulated maximum (C_{max}) and trough (C_{trough}) plasma concentrations, and area under the curve (AUC) values for the three dosing regimens commonly used for treatment of antenatal corticosteroids as shown in Fig. 8. The values are reported as median [Q5, Q95] of N=200 simulated subjects.

Regimen	C_{max}, ng/mL	C_{trough}, ng/mL	AUC_{0-48}, ng/mL·h	AUC_{0-72}, ng/mL·h
DEX 6 mg IM 4xBID	71.6 [69.6,74.0]	33.1 [31.0,35.0]	2366 [1869, 2977]	2521 [1948, 3272]
BET 12 mg IM 2xQD	144.2 [139.9,148.8]	26.4 [25.3,27.5]	3594 [2881,4454]	3861 [3073,4822]
CEL 12 mg IM 2xQD	77.4 [75.0,80.4]	17.1 [16.4,17.9]	2021 [1620, 2508]	2342 [1862,2929]

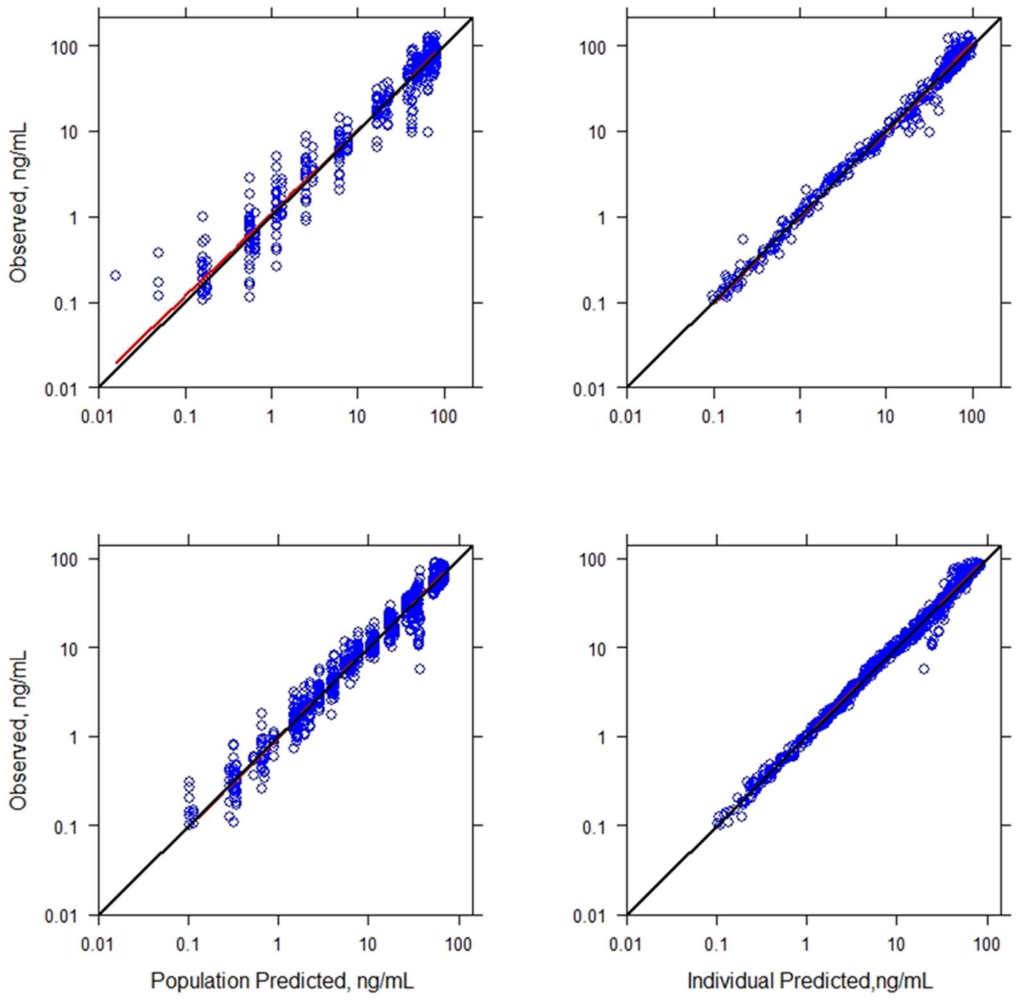

Fig. 1S. Observed versus predicted diagnostic plots for DEX (upper panels) and BET (lower panels) plasma concentrations. The red lines are the LOESS curves.

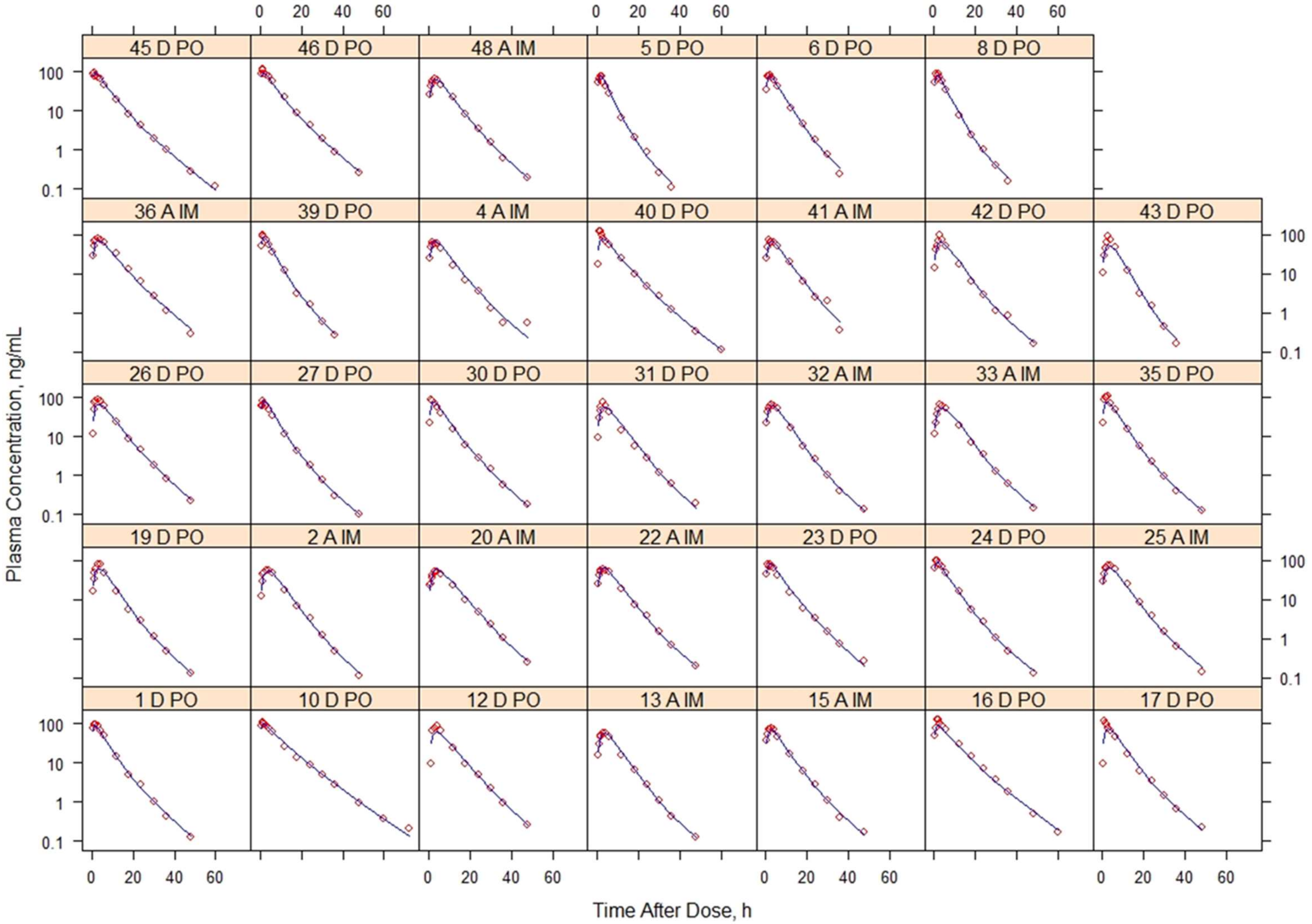

Fig. 2S. Individual plots of DEX observed (symbols) and predicted (lines) plasma concentrations vs time for DEX-IM and DEX-PO administered in sequences AB, BA, CD, DC, DE, and ED. Subject numbers, sequence, and dosing routes are indicated

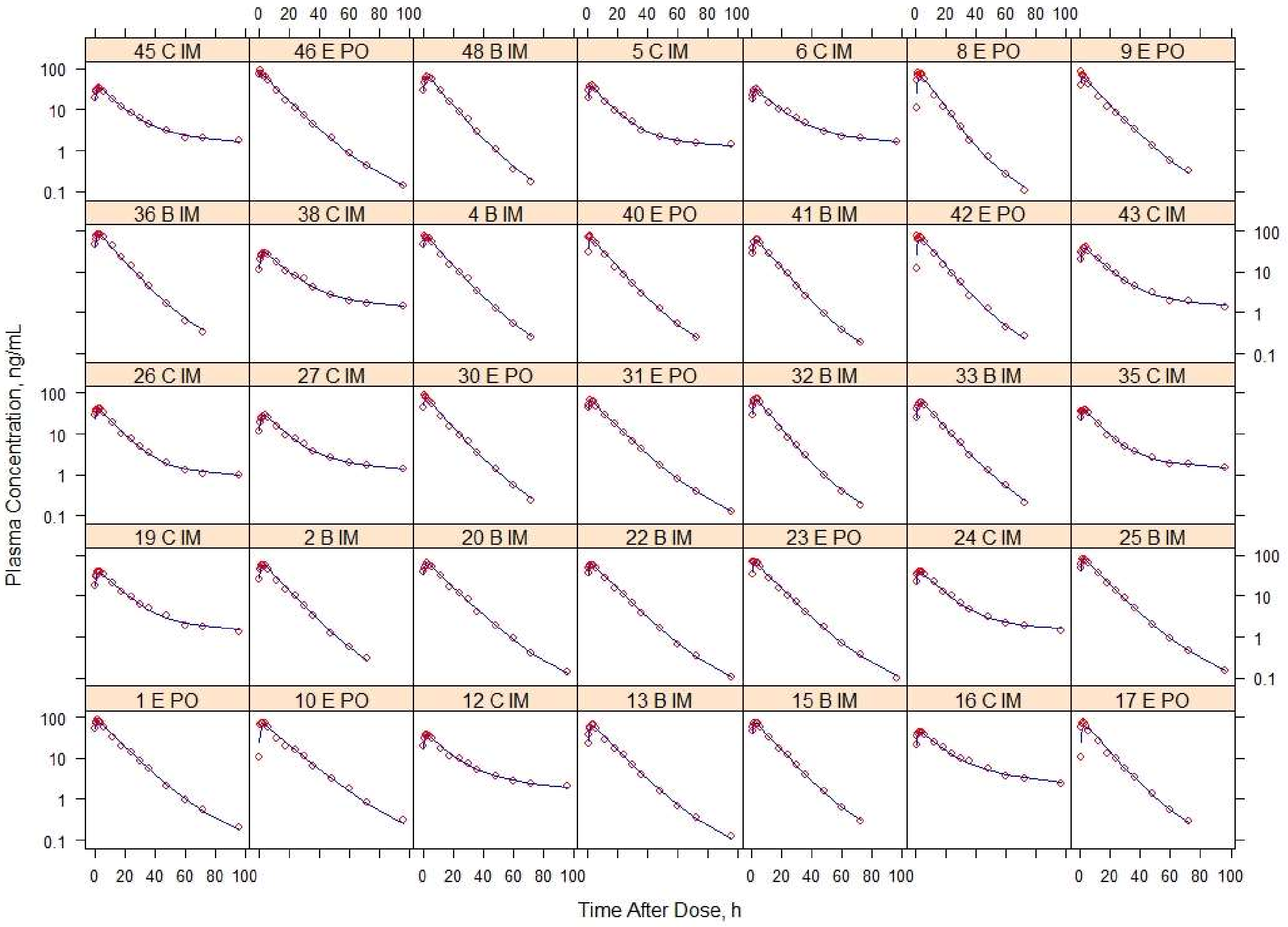

Fig. 3S. Individual plots of BET observed (symbols) and predicted (lines) plasma concentrations vs time for BET-IM, BET-PO and BET-PA administered in sequences AB, BA, CD, DC, DE, and ED. Subject numbers, sequence, and dosing routes are indicated.

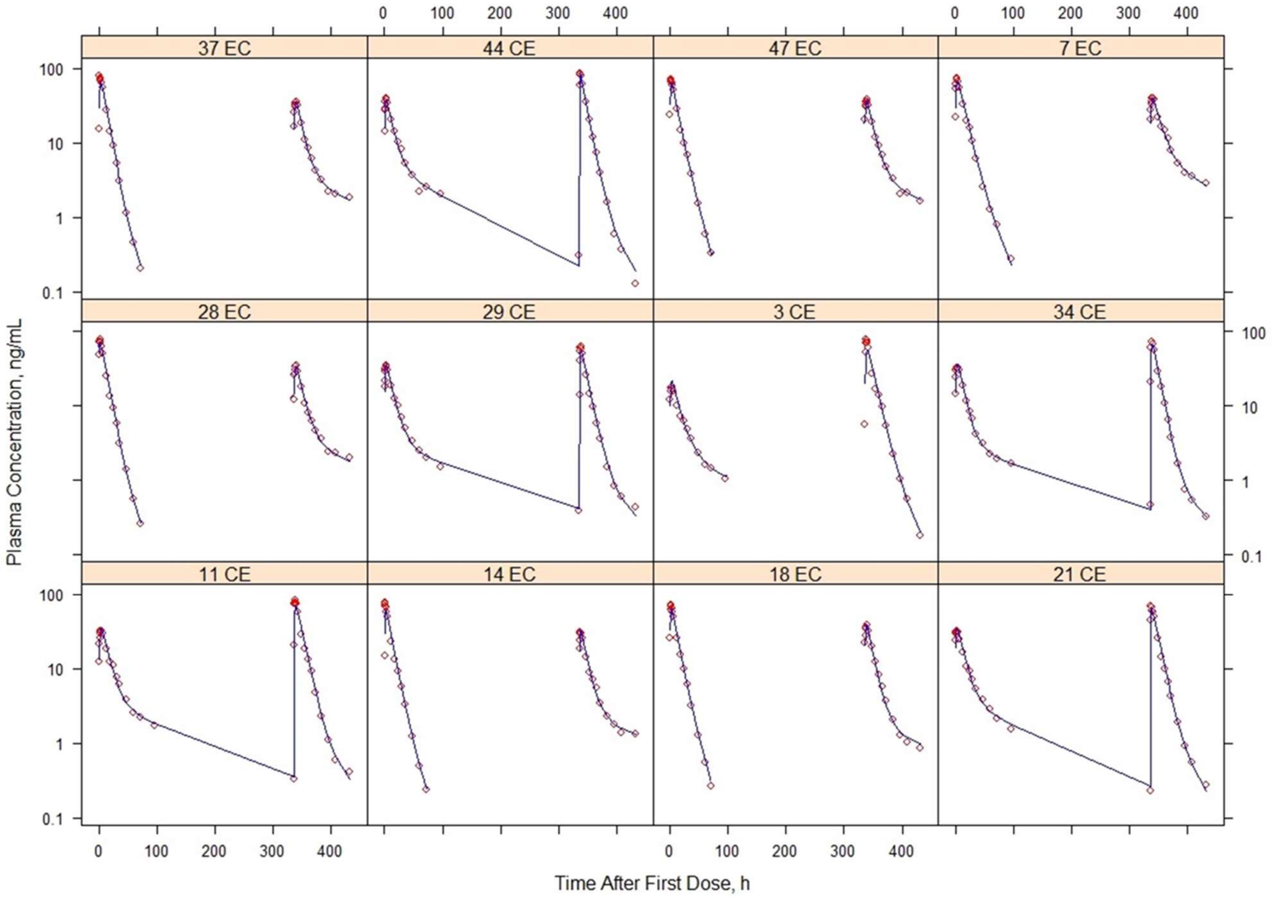

Fig. 4S. Individual plots of BET observed (symbols) and predicted (lines) plasma concentrations vs time for BET PO and BET PA administered in sequences CE and EC. Subject numbers, sequence, and dosing routes are indicated.
